# The Effect of Diagnostic Imaging on Surgical Treatment Planning in Diseases of the Thymus

**DOI:** 10.1155/2017/9307292

**Published:** 2017-01-10

**Authors:** Aurel Ottlakan, Bernadett Borda, Zita Morvay, Aniko Maraz, Jozsef Furak

**Affiliations:** ^1^Faculty of Medicine, Department of Surgery, University of Szeged, Szokefalvi-Nagy Bela Street 6, Szeged 6720, Hungary; ^2^Faculty of Medicine, Department of Radiology, University of Szeged, Szokefalvi-Nagy Bela Street 6, Szeged 6720, Hungary; ^3^Faculty of Medicine, Department of Oncology, University of Szeged, Szokefalvi-Nagy Bela Street 6, Szeged 6720, Hungary

## Abstract

Accurate imaging of the thymus is essential in the diagnosis and surgical treatment of both neoplastic and nonneoplastic conditions. Imaging of the thymus is a rather complex task, which affects both initial diagnosis and further surgical treatment planning. Imaging techniques include a wide armamentary of possibilities, from the most frequently used computed tomography (CT) to 18-fluorodeoxyglucose positron emission tomography- (18-FDG-PET-) CT and chemical shift magnetic resonance imaging (CS-MRI). In cases where surgical treatment is involved diagnostic imaging is of pivotal importance, not only in distinguishing benign from malignant disease but also in making a way among subtypes of thymic conditions. The article presents a current review of the advantages and backdrops of different imaging techniques used in the diagnosis of benign and malignant thymic conditions, with emphasis on differential imaging of thymic hyperplasia (TH), ectopic thymic tissue (ETT), and thymic epithelial tumors (TETs), with special attention to the importance of MR imaging according to the new TNM classification of thymic epithelial tumors.

## 1. Introduction

Accurate diagnostic imaging is of great importance in the treatment management of thymic pathologies. Regarding thymectomy, there are three important topics which are significant during a discussion between a radiologist and a thoracic surgeon: (1) differentiating between thymic hyperplasia (TH) and thymoma (THA), (2) deciding whether a possible thymoma invades surrounding tissues, and (3) declaring the presence of ectopic thymic tissue (ETT) around the thymus. The answers given by the radiologist absolutely predict the decision of the thoracic surgeon. The diagnosis of THA alone is an absolute indication of thymectomy, though differing thymic abnormalities such as TH without myasthenia gravis (MG) should not be treated surgically. In cases of THA or thymic carcinoma (TC) treatment planning is based on imaging results. Thus dilemmas arise, whether it is possible to carry out a primary resection or neoadjuvant treatment should be the first step and how can the extent of resection be defined? In cases of MG it is very important to evaluate any lesion in the mediastinum which could raise the suspicion of ETT, because surgical removal of these lesions can highly improve the success rate of a thymectomy. Our article deals with the role of preoperative imaging and its effects on surgical decision making in the most relevant conditions of the thymus.

## 2. Thymic Hyperplasia or Thymoma

The rate of unnecessary or nontherapeutic thymectomies carried out according to a previous CT diagnosis is 43.8% (of which 17.1% are thymic hyperplasia cases) which emphasizes the importance of accurate preoperative diagnosis of thymic lesions [1]. This is an important question for a thoracic surgeon because presence of THA means an absolute indication for surgery, while TH without MG is to be treated nonsurgically. Preoperative imaging must have a definitive answer to this dilemma which greatly influences surgical decision making. Although chest CT is considered to be the routinely used modality in the imaging of thymic lesions, in the differentiation between TH and THA, in cases where preoperative diagnosis is ambiguous, MR imaging should be performed [2, 3].

### 2.1. MRI

In recent years MRI has integrated two important sequences into its armamentary, namely, fat suppression and chemical shift imaging. Due to the presence of both fat and water, thymic tissue around the tumor will show signal intensity loss on opposed-phase imaging; hence chemical shift imaging can be useful in pointing out the tumor margin and rate of invasion. In older age thymic tissue has already almost completely been replaced by fat; therefore in older patients fat suppression imaging should be the method of choice to define exact tumor margins. With the use of fat suppression-MRI (FS-MRI) and chemical shift-MRI (CS-MRI), diagnosis can be more accurate. CS-MRI is able to differentiate TH from THA [2] and according to the study of Li et al., differentiating between these two entities is much more accurate using FS-MRI and CS-MRI. In case of young patients CS-MRI proved to be better than CT, conventional MRI, or fat suppression imaging in distinguishing TH from THA and locating the exact tumor margin. In elderly patients, fat suppression imaging is the right choice, especially in cases of THA [3]. Priola et al. analyzed the qualitative and quantitative values of CT and CS-MRI in differentiating between THA and lymphoid thymic hyperplasia (lTH) among 83 patients with MG. In terms of qualitative values, the accurate diagnosis was achieved in 86.7% and 96.4% of cases for CT and MRI, respectively. Specificity and positive predictive value (PPV) showed significant differences between the two modalities (*p* = 0.048 versus 0.04), proving MRI to be more reliable [4].

### 2.2. Scintigraphy and PET-CT

*Thallium 201 (201Tl) scintigraphy* is also useful in the evaluation of thymic lesions. It represents cellular metabolic activity, regional blood flow, and the number of viable cells and is helpful in distinguishing between normal thymus (NT), TH, and THA in cases of MG. In cases of TH and THA a higher uptake was seen in different periods compared to the ones seen in case of the NT [5]. The disadvantages of this modality (high cost, low throughput, and irradiation to the patient) precluded it from being widely used in imaging of the thymus.

*FDG-PET* is more accurate in determining the metabolic and functional features than the morphologic characteristics of neoplasms with the possible recognition of residual or recurrent disease. In the study of El-Bawab et al., the role of FDG-PET was investigated among different thymic lesions (TH, THA, and TC). The ranges of standardized uptake value (SUV) were seen between 0.7–2.5 and 3.1–6.1 in TH and THA, respectively [6].

## 3. Thymoma

In cases where the diagnosis of THA is obvious and an absolute indication for thymectomy is obtained, the most important factor for the surgeon is the radio-clinicopathological data concerning the THA. The following questions arise. (1) Is the THA resectable, (2) does it infiltrate surrounding tissues so that neoadjuvant therapy should come first, (3) what is the rate of regression after neoadjuvant treatment, and (4) should the resection be extended? These questions can be answered after an extended and more meticulous imaging process with the adaptation of the newly proposed TNM classification. Relationship to surrounding tissues or organs (mainly great vessels and myocardium), the presence or lack of lymph node metastases (N1, N2), and involvement of pleural/pericardial sites should be investigated and mentioned.

### 3.1. Histological Classification, TNM, and Staging of Thymoma

The diagnosis and classification of thymic epithelial tumors (TET) have been a topic of argument for many years. TETs have been uniformly classified by the WHO in 1999, with emphasis on both clinical and functional aspects [7]. Type A tumors consist of rod-like epithelial cells without the presence of atypia or neoplastic lymphocytes. Type AB tumors resemble type A tumors but already represent centers of neoplastic lymphocytes. Type B tumors feature round-shaped epithelioid cells and are further divided into subtypes (B1, B2, and B3). Type C defines thymic carcinoma [8].

The recent uniform staging classification system was processed by the Thymic Domain of the Staging and Prognostic Factors Committee (TD-SPFC) in collaboration with IASLC and ITMIG [9]. The most popular and widely used Masaoka-Koga stage classification system (MK-SCS) [10] was integrated into the new one; however some significant modifications have also been introduced. These new changes also have an important aspect in terms of imaging. One of the most important changes is the elimination of a previously essential focus, on which a tumor is encapsulated or, by expanding beyond the border of the capsule, infiltrates the thymus and neighbouring fat. This important modification is based on the fact that all THAs are considered malignant, irrelevant of the presence or lack of a capsule. In terms of the TNM system, T1 has been divided into two subtypes, namely, T1a (without mediastinal pleural involvement) and T1b (involvement of the mediastinal pleura). In T2 cases the pericardium is involved, while in T3, the lung, brachiocephalic vein, superior vena cava, chest wall, phrenic nerve, and hilar pulmonary vessels are invaded. The aorta, great branches of the pulmonary artery, myocardium, trachea, or esophagus are involved in T4 tumors [9].

Preoperative imaging should point out the exact borders and presence or lack of invasion of THA concerning the above-mentioned tissues or organs. In resectable cases primary thymectomy is indicated, while in case of an unresectable tumor, neoadjuvant treatment should be applied.

An additional useful and important change is the verification of regional thymic lymph node levels. The N1 level involves the anterior nodes, while the N2 level is limited to the deep intrathoracic or cervical nodes [9, 11]. Lymph node metastases most frequently occur in the anterior mediastinum and can be proved in 2% of THAs, 27% of TCs, and 28% of neuroendocrine thymic tumors (NETT) [11]. The M category has been defined as M0, the tumor being limited to the primary mass, M1a with pericardial or pleural nodules and M1b showing nodules inside the lung parenchyma or being present in distant locations [9]. The N and M status of THAs should be mentioned during imaging.

### 3.2. Imaging Features of Thymic Tumors

In the diagnostic imaging of thymic epithelial tumors (TETs) and in the differentiation between various WHO subtypes, CT imaging plays a key role [12, 13]. In the study by Tomiyama et al., CT characteristics of 53 primary thymic tumors were investigated retrospectively, according to the WHO classification. Type A tumors were more frequently described by smooth contour and a round shape, than TCs which more often appeared with an irregular contour. Curvilinear calcifications were seen in 20% of cases, usually meaning poor prognosis [14]. Mediastinal and hilar lymphadenopathy was found in nearly half of TC cases [15]. According to the study conducted by Sadohara et al. on distinguishing between subtypes of THAs, they found that the likelihood of invasion seen on CT was relatively low in low-risk THAs, intermediate in high-risk THAs, and highest in TCs. Irregular contour, necrotic or cystic component, heterogeneous enhancement, lymphadenopathy, and great vessel invasion were helpful in differentiating low- or high-risk THAs from TCs [16].

In the study conducted by Zhu et al. which focuses on surgical outcomes, the rate of neoadjuvant treatment was 6% in case of THAs (B2-B3-TC) with a 15% rate of incomplete resection [17]. The study by Hayes et al. described that in 87% of incomplete resections, on preoperative CT peritumoral fat was missed, compared to 66% rate in operable cases. In cases of THAs where more than 50% circumference of the adjacent vessel was involved, it was impossible to carry out the resection [13]. The study by Korst et al. states that CT and PET-CT are safe and useful methods in acquiring diagnosis and deciding resectability after neoadjuvant chemoradiotherapy. The rate of resection was 77% and the debulking rate was only 5% after neoadjuvant therapy. With the help of this method the rate of regression after neoadjuvant treatment can be defined since the median SUV max decreased with 45% after neoadjuvant chemoradiotherapy [18]. Octreotide scintigraphy is useful in the diagnosis of THA and its metastases but it is not positive in TH [19, 20].

MRI can be helpful in differentiating among THA subtypes and can predict the aggressivity of the disease. The presence of a septum within the tumor is highly suggestive of THA. On MR imaging, the tumors showing smooth contour, almost complete capsule, septum within the tumor, and homogenous enhancement are more likely to be low-risk, than high-risk THAs or TCs. Sadohara's study states that MRI is more accurate in detecting a capsule, septum, or a haemorrhage than CT [16].

It has been declared that diffusion weighted imaging (DWI) with the measurement of the apparent diffusion coefficient (ADC) can be helpful to distinguish between benign and malignant thymic lesions [21]. In addition, an extremely useful aspect of this modality is the ability to follow the rate of regression in case of THAs after neoadjuvant chemotherapy [21].

## 4. Role of Imaging in Myasthenia Gravis (MG)

The thymus is definitely involved in the pathogenesis of MG. The incidence of thymic pathologies occurring among MG patients is roughly 75% [22, 23]: with TH occurring in 60–77% and THA in 15–30% of cases [24]. In case of MG thymectomy is necessary when the disease is accompanied by THA; however when TH alone is present in MG patients, thymectomy can be recommended, but it is not mandatory. In the current clinical practice preoperative imaging of ETT is not a routine procedure, although a number of MRI and PET-CT reports state that in numerous cases ETT can be detected on the neck or in the mediastinum. In terms of MG thymectomies it would be extremely advantageous if some information could be routinely obtained on imaging about the localization of ETT.

Principles of surgical treatment of MG include complete removal of the thymus with perithymic fat and possible ETT. In MG cases usually two questions arise in terms of surgical treatment. (1) Does the patient have THA? If the answer is yes, it is an absolute indication for thymectomy. (2) Is there any amount of ETT or “abnormal fat” around the thymus or in the mediastinum? In case ETT remains in the mediastinum or on the neck, the postoperative improvement of MG is significantly worse.

### 4.1. Embryology of Ectopic Thymic Tissue

During its development, thymic primordia arise from the third and fourth pharyngeal pouches, become cylindrical, form the thymopharyngeal ducts, and descend into the anterior mediastinum. On the 8th gestational week, the thymic primordia fuse at their lower poles and on the 14th to 16th weeks, the thymus further differentiates into cortical and medullary components [25].

### 4.2. Prevalence and Imaging Techniques of Ectopic Thymic Tissue

ETT may be found in various locations according to the path of descent [25] and anywhere in the mediastinum, mainly around the thymus. According to the study conducted by Zieliński et al. [26] among 100 nonthymomatous patients with MG, ectopic thymic foci were identified in 71% of patients with the highest incidence in perithymic fat (37%) and at the site of the aortopulmonary window (33%), in the cervical region (10%), in the right and left pericardiophrenic fat (7% each), and in the aortocaval groove (4%). Imaging modalities of ETT include ultrasound (US) in infants, bearing the advantages of short examination time, high resolution, and the lack of ionizing radiation [27]. US features of ETT include echogenic linear structures, which may refer to connective tissue septa [28]. In terms of FDG-PET, a study by El-Bawab et al. confirmed that FDG-PET was insufficient in the detection of ETT [6]. On MRI, ETT appears homogenous, isointense, or slightly hyperintense compared with muscle on T1 weighted images and hyperintense on T2 weighted images [29].

## 5. Surgical Point of View of Thymectomy

All THAs are considered malignant, due to their invasive and metastatic potential, and thus should be surgically resected in every case. In terms of surgical or nonsurgical treatment planning, precise staging is one of the most important factors. Preoperative imaging should mention T, N, and M status of the THA with special attention to perithymic invasion. Performing a complete resection of not only the gland itself but also surrounding tissues containing thymic cells and lymph nodes is of utmost importance. Incomplete resection is associated with a high-recurrence rate and poor prognosis. Throughout the years several surgical methods have been described for the resection of THAs. The traditional approach is the median sternotomy. The MIA (Minimally Invasive Approach) for resection of THAs has gained widespread popularity in recent years. These techniques include VATS thymectomy from both sides, cervical thymectomy, subxiphoidal thymectomy, uniportal thymectomy, and robotic resection. VATS thymectomy is a radical and expansively used minimally invasive technique in the successful removal of not only the gland but also surrounding thymic tissue [30]. In advanced THA or TC cases extended resections are recommended in combination with (neo) adjuvant chemo-/radiotherapy [20].

## 6. Summary and Outlook

Imaging evaluation of the thymus comprises many obstacles, mainly due to the fact that there are changes in size, shape, consistency, and amount of fat with age to the organ. Precise diagnosis and differentiation between each thymic condition through imaging are essential for ideal surgical treatment planning and avoiding overtreatment. While CT remains the cornerstone of thymic imaging, MRI evolves as a useful problem-solving modality for evaluation of various thymic conditions and may remarkably support CT in everyday clinical practice, especially in cases accompanied by MG in combination with different types of the THAs or TC. CT combined with PET imaging can be effectively used in the diagnosis of advanced THAs or TC, with control of regression after neoadjuvant treatment, thus facilitating the rate of surgical success. MRI is superior to CT in distinguishing normal and hyperplastic thymus from THA. With the adding of chemical shift sequence, MRI maintains a higher accuracy in distinguishing THAs from TH which is essential in the algorithm of treatment planning and deciding whether surgery is needed. In terms of neoplastic conditions, MRI proved to be an accurate modality in differentiating high- and low-risk thymomas and can be helpful in separating THA from TC. Distinguishing among various thymoma subtypes on imaging is fundamental for further treatment planning (preoperative chemo- and radiotherapy or primary surgical resection) and achieving total remission. It would be beneficial if the new proposed TNM and regional N-stage classification for TETs could be more accurately described by preoperative imaging similarly to the current N-stage system for lung cancer, thus contributing to more precise clinical classification. Treating patients with MG is one of the mainstays of thymic surgery. Total removal of the thymus and the resection of ETT in typical locations (perithymic fat, aortopulmonary window, cervical region, right and left pericardiophrenic fat, and aortocaval groove) are of paramount importance in banishing MG. Preoperative imaging could be extremely helpful in discovering possible ectopic thymic foci.

## Abbreviations

CT: Computed tomography
MRI: Magnetic resonance imaging
CS-MRI: Chemical shift magnetic resonance imaging
FS-MRI: Fat suppression magnetic resonance imaging
DW-MRI: Diffusion weighted magnetic resonance imaging
18-FDG-PET: 18-fluorodeoxyglucose positron emission tomography
201Tl: Thallium 201
US: Ultrasound
SUV: Standardized uptake value
PPV: Positive predictive value
ADC: Apparent diffusion coefficient
SSI: Signal intensity index
NT: Normal thymus
TH: Thymic hyperplasia
lTH: Lymphoid thymic hyperplasia
TET: Thymic epithelial tumor
THA: Thymoma
TC: Thymic carcinoma
NETT: Neuroendocrine thymic tumor
ETT: Ectopic thymic tissue
MG: Myasthenia gravis
MIA: Minimally invasive approach
VATS: Video-assisted thoracoscopic surgery
WHO: World Health Organization
IASLC: International Association for the Study of Lung Cancer
ITMIG: International Thymic Malignancy Interest Group
MK-SCS: Masaoka-Koga stage classification system
TD-SPFC: Thymic Domain of the Staging and Prognostic Factors Committee.

## References

[1] Ackman J. B., Verzosa S., Kovach A. E. (2015). High rate of unnecessary thymectomy and its cause. Can computed tomography distinguish thymoma, lymphoma, thymic hyperplasia, and thymic cysts?. *European Journal of Radiology*.

[2] Popa G. A., Preda E. M., Scheau C., Vilciu C., Lupescu I. G. (2012). Updates in MRI characterization of the thymus in myasthenic patients. *Journal of Medicine and Life*.

[3] Li K., Yang D., Hou S., Xu X., Gong T., Chen H. (2014). Chemical shift and fat suppression magnetic resonance imaging of thymus in myasthenia gravis. *Canadian Journal of Neurological Sciences*.

[4] Priola A. M., Priola S. M., Gned D., Giraudo M. T., Fornari A., Veltri A. (2016). Comparison of CT and chemical-shift MRI for differentiating thymoma from non-thymomatous conditions in myasthenia gravis: value of qualitative and quantitative assessment. *Clinical Radiology*.

[5] Higuchi T., Taki J., Kinuya S. (2001). Thymic lesions in patients with myasthenia gravis: characterization with thallium 201 scintigraphy. *Radiology*.

[6] El-Bawab H., Al-Sugair A. A., Rafay M., Hajjar W., Mahdy M., Al-Kattan K. (2007). Role of flourine-18 fluorodeoxyglucose positron emission tomography in thymic pathology. *European Journal of Cardio-Thoracic Surgery*.

[7] Rosai J., Sobin L. H. (1999). *Histological Typing of Tumours of the Thymus: International Histological Classification of Tumours*.

[8] Detterbeck F. C., Parsons A. M. (2004). Thymic tumors. *Annals of Thoracic Surgery*.

[9] Detterbeck F. C., Stratton K., Giroux D. (2014). The IASLC/ITMIG thymic epithelial tumors staging project: proposal for an evidence-based stage classification system for the forthcoming (8th) edition of the TNM classification of malignant tumors. *Journal of Thoracic Oncology*.

[10] Masaoka A., Monden Y., Nakahara K., Tanioka T. (1981). Follow-up study of thymomas with special reference to their clinical stages. *Cancer*.

[11] Kondo K., Monden Y. (2003). Lymphogenous and hematogenous metastasis of thymic epithelial tumors. *Annals of Thoracic Surgery*.

[12] McErlean A., Huang J., Zabor E. C., Moskowitz C. S., Ginsberg M. S. (2013). Distinguishing benign thymic lesions from early-stage thymic malignancies on computed tomography. *Journal of Thoracic Oncology*.

[13] Hayes S. A., Huang J., Plodkowski A. J. (2014). Preoperative computed tomography findings predict surgical resectability of thymoma. *Journal of Thoracic Oncology*.

[14] Qu Y.-J., Liu G.-B., Shi H.-S., Liao M.-Y., Yang G.-F., Tian Z.-X. (2013). Preoperative CT findings of thymoma are correlated with postoperative Masaoka clinical stage. *Academic Radiology*.

[15] Tomiyama N., Johkoh T., Mihara N. (2002). Using the World Health Organization classification of thymic epithelial neoplasms to describe CT findings. *American Journal of Roentgenology*.

[16] Sadohara J., Fujimoto K., Müller N. L. (2006). Thymic epithelial tumors: comparison of CT and MR imaging findings of low-risk thymomas, high-risk thymomas, and thymic carcinomas. *European Journal of Radiology*.

[17] Zhu L., Zhang J., Marx A., Weiss C., Fang W. (2016). Clinicopathological analysis of 241 thymic epithelial tumors—experience in the Shanghai Chest Hospital from 1997–2004. *Journal of Thoracic Disease*.

[18] Korst R. J., Bezjak A., Blackmon S. (2014). Neoadjuvant chemoradiotherapy for locally advanced thymic tumors: a phase II, multi-institutional clinical trial. *The Journal of Thoracic and Cardiovascular Surgery*.

[19] Guidoccio F., Grosso M., Maccauro M. (2011). Current role of 111In-DTPA-octreotide scintigraphy in diagnosis of thymic masses. *Tumori*.

[20] Ried M., Marx A., Götz A., Hamer O., Schalke B., Hofmann H. (2016). State of the art: diagnostic tools and innovative therapies for treatment of advanced thymoma and thymic carcinoma. *European Journal of Cardio-Thoracic Surgery*.

[21] Seki S., Koyama H., Ohno Y. (2014). Diffusion-weighted MR imaging vs. multi-detector row CT: direct comparison of capability for assessment of management needs for anterior mediastinal solitary tumors. *European Journal of Radiology*.

[22] Scorsetti M., Leo F., Trama A. (2016). Thymoma and thymic carcinomas. *Critical Reviews in Oncology/Hematology*.

[23] Drachman D. B. (1994). Myasthenia gravis. *New England Journal of Medicine*.

[24] Pirronti T., Rinaldi P., Batocchi A. P., Evoli A., Di Schino C., Marano P. (2002). Thymic lesions and myasthenia gravis. Diagnosis based on mediastinal imaging and pathological findings. *Acta Radiologica*.

[25] Nasseri F., Eftekhari F. (2010). Clinical and radiologic review of the normal and abnormal thymus: pearls and pitfalls. *Radiographics*.

[26] Zieliński M., Kuzdzał J., Szlubowski A., Soja J. (2004). Transcervical-subxiphoid-videothoracoscopic ‘maximal’ thymectomy—operative technique and early results. *The Annals of Thoracic Surgery*.

[27] Ozel A., Akdur P. O., Celebi I., Karasu R., Yilmaz B., Basak M. (2015). Ectopic cervical thymus as a rare cause of pediatric neck mass: the role of ultrasound and MRI in the diagnosis. Case report. *Medical Ultrasonography*.

[28] Han B. K., Suh Y.-L., Yoon H.-K. (2001). Thymic ultrasound. I. Intrathymic anatomy in infants. *Pediatric Radiology*.

[29] Slovis T. L., Meza M., Kuhn J. P. (1992). Aberrant thymus: MR assessment. *Pediatric Radiology*.

[30] Raza A., Woo E. (2016). Video-assisted thoracoscopic surgery versus sternotomy in thymectomy for thymoma and myasthenia gravis. *Annals of Cardiothoracic Surgery*.

